# Multicentral Retrospective Analysis of Venetoclax-Based Treatments in AML and MDS: A Real-World Study by the Turkish Hematology Network Group

**DOI:** 10.3390/medicina60101623

**Published:** 2024-10-04

**Authors:** Ibrahim Halil Acar, Muzeyyen Aslaner Ak, Gulsah Akyol, Taha Ulutan Kars, Yildiz Ipek, Ayse Uysal, Figen Atalay, Aysun Senturk Yikilmaz, Omer Ekinci, Idris Ince, Birgul Onec, Hakan Keski, Mufide Okay Ozgeyik, Sebnem Izmir Guner, Esra Terzi Demirsoy, Oktay Bilgir, Birol Guvenc

**Affiliations:** 1 Hematology Clinic, Osmaniye State Hospital, 80000 Osmaniye, Türkiye; halil_acar_63@hotmail.com; 2 Department of Hematology, Faculty of Medicine, Zonguldak Bulent Ecevit University, 67100 Zonguldak, Türkiye; 3 Division of Hematology, Department of Internal Medicine, Faculty of Medicine, Erciyes University, 38030 Kayseri, Türkiye; 4 Hematology Clinic, Konya City Hospital, Health Sciences University, 42020 Konya, Türkiye; 5 Hematology Clinic, Istanbul Kartal Dr. Lutfi Kirdar City Hospital, 34865 Istanbul, Türkiye; 6 Division of Hematology, Department of Internal Medicine, Faculty of Medicine, Fırat University, 23200 Elazıg, Türkiye; 7 Division of Hematology, Department of Internal Medicine, Faculty of Medicine, Yeditepe University, 34718 Istanbul, Türkiye; f_noyan@yahoo.com; 8 Hematology Clinic, Denizli State Hospital, 20010 Denizli, Türkiye; 9 Department of Adult Bone Marrow Transplantation, Kolan International Hospital, 34384 Istanbul, Türkiye; 10 Hematology Clinic, Gaziantep City Hospital, 27470 Gaziantep, Türkiye; 11 Division of Hematology, Department of Internal Medicine, Faculty of Medicine, Duzce University, 81010 Duzce, Türkiye; 12 Hematology Clinic, Umraniye Training and Research Hospital, Health Sciences University, 34760 Istanbul, Türkiye; 13 Hematology Clinic, Eskisehir City Hospital, Health Sciences University, 26080 Eskisehir, Türkiye; mufide_okay@yahoo.com; 14 Department of Adult Bone Marrow Transplantation, Hisar Hospital Intercontinental, 34768 Istanbul, Türkiye; 15 Division of Hematology, Department of Internal Medicine, Faculty of Medicine, Kocaeli University, 41001 Kocaeli, Türkiye; 16 Hematology Clinic, Bozyaka Training and Research Hospital, Health Sciences University, 35170 Izmir, Türkiye; 17 Department of Hematology, Faculty of Medicine, Adana Çukurova University, 01790 Adana, Türkiye

**Keywords:** acute myeloid leukemia, myelodysplastic syndrome, venetoclax, treatment outcome, malignancy

## Abstract

*Background and Objectives:* Acute myeloid leukemia and myelodysplastic syndrome are both clonal hematologic malignancies that primarily affect older adults. Current treatments for AML/MDS are both limited in number and efficacy. This study aims to evaluate venetoclax-based therapies in AML/MDS, focusing on overall survival and recurrence-free survival rates, and to expand real-world data on its use. *Materials and Methods:* Clinical and laboratory data on patients with AML/MDS aged 18≥ treated with venetoclax between January 2019 and July 2022 were included. Survival analysis was calculated based on the period from 2019 to December 2023. *Results:* A total of 161 AML and 40 patients with MDS were included. The median age was 63.53 ± 15.30 years for AML and 70.12 ± 10.21 years for MDS. In both groups, over 55% are male. A total of 77.6% of patients with AML and 75% of patients with MDS received treatment prior to venetoclax. Venetoclax was administered in combination with azacitidine to 84.5% of AML and 67.5% of MDS. The relapse rate in AML is approximately 15%. Overall, the 2-year survival rate is 46% and 18.73 months. The overall CR/CRi rate for patients with AML is 49.1%, while for patients with MDS, it is 50%. The 2-year survival rate for patients with MDS is 52.7%. The 2-year RFS rate was 75.5% for AML and 90.9% for MDS. The relapse rate in AML is approximately 15%. The percentage of adverse events leading to treatment discontinuation among those with grade 3–4 toxicity is low; 26.7% for AML (*n* = 43) and 15% for MDS (*n* = 6). *Conclusions:* Our real-world data demonstrate that venetoclax has the potential to improve overall survival rates when used in combination with HMAs and supports its use in patients with AML/MDS.

## 1. Introduction

Acute myeloid leukemia (AML) and myelodysplastic syndrome (MDS) are both clonal hematologic malignancies that primarily affect older adults. AML is characterized by the clonal proliferation of myeloid blast cells in peripheral blood, bone marrow, and other tissues, with a median age at diagnosis of 67 years according to the Surveillance, Epidemiology, and End Results (SEER) Program of the National Cancer Institute (NCI) [1]. This aggressive leukemia subtype has a notably low 5-year survival rate of 31.9%, making it the leukemia type with the highest mortality rate [1]. Similarly, MDS is a clonal bone marrow neoplasm characterized by morphological dysplasia in hematopoietic cells, peripheral cytopenias, ineffective hematopoiesis, and recurrent genetic abnormalities [2]. It also carries an increased risk of progression to AML [3]. MDS typically manifests in older individuals, with a median age at diagnosis often ≥65 years [4].

Allogeneic hematopoietic stem cell transplantation (HSCT) is currently considered the sole potentially curative approach for numerous patients diagnosed with acute AML and MDS [5]. Due to the low efficacy and high toxicity of conventional induction chemotherapy protocols, their use as a bridging approach to HSCT is often not feasible for many patients with AML. Venetoclax, a BH3 mimetic and small molecule inhibitor of the anti-apoptotic protein B-cell lymphoma 2 (BCL2), was approved by the Food and Drug Administration (FDA) in the United States in November 2018 for newly diagnosed patients with AML who are unable to tolerate intensive chemotherapy [6].

Venetoclax was initially evaluated in relapsed/refractory AML, showing an overall response rate (ORR) of 19% and suggesting its potential use as a single agent due to its good safety profile [7]. Preclinical studies have highlighted the synergistic effects of venetoclax with both hypomethylating agents (HMAs) and azacytidine, cytarabine, or decitabine. Multi-center phase I/II clinical trials have been conducted to evaluate venetoclax in combination with LDAC or HMAs for newly diagnosed patients with AML who are ineligible for intensive chemotherapy [8,9,10,11]. Studies have shown that azacitidine and venetoclax induce cell death in AML-derived cell lines [10,12]. These combination regimens lead to significantly different treatment outcomes compared to single-agent LDAC or HMAs. Moreover, long-term use of venetoclax has been implicated in causing myelosuppression leading to infections or other adverse events associated with cytopenia. Venetoclax can also lead to tumor lysis syndrome (TLS), necessitating appropriate preventive measures [4].

The primary objective of this study is to investigate the clinical outcomes of patients treated with venetoclax in AML/MDS, focusing on median overall and disease-free survival rates. Given the current lack of comprehensive knowledge regarding the use of venetoclax, expanding real-world data on its application is expected to provide valuable insights for future clinical trials.

## 2. Materials and Methods

This study is a retrospective, multicenter, observational investigation conducted in accordance with guidelines from local institutional research committees and principles outlined in observational studies on drug guidelines. Ethical approval has been obtained from the Non-Interventional Clinical Research Ethics Committee of Çukurova University (3 June 2022/Project Number: 123). In addition, all participating researchers who are members of the Turkish Hematology Network Group have received approval from the relevant departments of their hospitals or universities for the use of retrospective data.

Between January 2019 and July 2022, patients aged 18 ≥ years with AML/MDS treated with venetoclax were included. Clinical and laboratory data of patients were obtained from the databases of centers. Demographic and clinical data were comprehensively reviewed, and patients’ hematologic, biochemical, cytogenetic, and molecular analyses, along with timing, dosage, and duration of venetoclax treatment, as well as other chemotherapeutic and stem cell transplantations, were recorded. Patients’ records were stored in a database by using an electronic case report form (e-CRF) through the Turkish Hematology Network Group’s digital platform. Survival analysis was calculated based on the period from 2019 to December 2023.

Inclusion criteria were confirmed MDS diagnosis by cytological or histological examination according to WHO 2016 classification, confirmed relapsed/refractory or newly diagnosed AML diagnosis according to WHO 2016 classification, adequate renal and hepatic function (ALT/AST/Bilirubin ≤ 2.5 × upper limit of normal), at least one cycle of venetoclax treatment received, and sufficient performance status (ECOG 0–3, life expectancy > 3 months). Exclusion criteria included acute promyelocytic leukemia, known drug hypersensitivity, active viral infections including hepatitis B and C, human immunodeficiency virus (HIV), acquired immunodeficiency syndrome (AIDS), and severe acute respiratory syndrome coronavirus 2 (SARS-CoV-2), as well as pregnancy or breastfeeding.

Different responses to treatment, remission and relapse (hematologic/molecular) statuses, mortality and timelines, and causes of mortality were obtained from medical records. The definitions of the variables of interest are based on current European LeukemiaNet (ELN) recommendations for diagnosis and treatment of AML in adults [4]. Relapse was defined as more than 5% blasts in bone marrow aspirate material after complete remission, and extramedullary disease was defined as histologically confirmed blast cell infiltration outside the bone marrow in a patient with complete remission.

Definition of remission, response, and endpoints remission criteria were defined per the International Working Group (IWG) response criteria in myelodysplasia [13]. ORR was defined as the percentage of patients achieving CR, mCR, or PR. Duration of response (DOR) was defined as the time from when a patient achieved a CR/mCR until disease progression, relapse, or death. Overall survival (OS) was defined as the time from starting combination therapy to the time of death. Cytogenetic remission was defined as the abnormalization of abnormal cytogenetics at the time of remission. HMA failure was defined as having received at least four cycles of decitabine or six cycles of azacitidine prior to starting venetoclax [13].

After a 3-day escalating dose titration in the first cycle (100–200–400 mg), patients receiving venetoclax at a dose of 400 mg/day (first dose level) were included in the study. Treatment for AML is administered in cycles of 28 days each, repeated continuously based on response to therapy. In MDS, treatment starts at a dose of 400 mg and continues for durations of 14–21 or 28 days per cycle. In cases of myelosuppression, treatment is administered at the first dose level every 21 days initially, followed by subsequent cycles at either 21 or 14-day intervals. In cases of significant cytopenia, dose reduction to the third level occurs over 7 to 10 days.

The Venetoclax dose administered during the induction phase ranged from 100 mg to 600 mg, titrated over 3 days. Following remission, venetoclax was continued at doses ranging from 100 mg to 600 mg. In MDS treatment, venetoclax was administered for durations of 14 days, 21 days, or 28 days within each 28-day cycle. Venetoclax was used in monotherapy, in combination with azacitidine, decitabine, low-dose cytarabine (LDAC), and other agents. Routine blood counts, bone marrow aspiration, and biopsy results were used to assess hematologic response after each cycle (1st, 2nd, 3rd, 4th, and 6th cycles).

Adverse events were termed and graded retrospectively according to the Common Terminology Criteria for Adverse Events (CTCAE) version 4.03 [14]. Clinical and laboratory data regarding adverse events and hospitalizations were obtained through examination of patients’ files.

Statistical analyses were performed using IBM SPSS Statistics for Windows, Version 25.0 (Statistical Package for the Social Sciences, IBM Corp., Armonk, NY, USA). Descriptive statistics were presented as *n* and % for categorical variables and as mean ± SD and median (min–max) for continuous variables. The Kaplan–Meier method was used to compare survival and RFS times among various clinical parameter groups. A *p*-value < 0.05 was considered statistically significant.

## 3. Results

A total of 161 AML and 40 patients with MDS from 16 centers were included. The median age was 63.53 ± 15.30 years for patients with AML and 70.12 ± 10.21 years for patients with MDS. Among patients with AML, 58.4% were male, and 57.5% of patients with MDS were male. De novo AML was in 71.4% (*n* = 115) of AML cases, and 28.6% (*n* = 46) had secondary AML. FLT3 mutations were absent in 89.4% (*n* = 144) of patients with AML. According to cytogenetic risk assessment, 57.8% (*n* = 93) of patients with AML were in the intermediate risk group. For patients with MDS, 82.5% (*n* = 33) had unclassified MDS with excess blasts–refractory anemia. A total of 25% (*n* = 10) of patients with MDS are classified in a high-risk group according to the IPSS R score (Table 1).

Comorbidities in patients with AML were observed as follows: 19.3% (*n* = 31) diabetes, 40.4% (*n* = 65) hypertension, 13.7% (*n* = 22) coronary artery disease (CAD), and 5.6% (*n* = 9) cancer, while no genetic syndromes were observed. In patients with MDS, 22.5% (*n* = 9) have diabetes, 55% (*n* = 22) have hypertension, and 15% (*n* = 6) have CAD. Cancer, genetic syndromes, and familial inheritance were not observed. Environmental exposure was noted in 2.5% (*n* = 4) of patients with AML, with pesticides observed in 50% (*n* = 2), and benzene and radiation exposure each observed in 25%. In patients with MDS, environmental exposure was detected in 7.5% (*n* = 3), with pesticides found in 75% and benzene in 25%. Autoimmune diseases were identified in 5.6% of patients with AML, with rheumatoid arthritis observed in 44.4% of these patients. No autoimmune diseases were observed in patients with MDS. PNH flare was positive in 1.2% of patients with AML and 7.5% of patients with MDS.

A total of 77.6% (*n* = 125) of patients with AML received treatment prior to venetoclax, and of these patients, 29.3% (*n* = 89) were administered cytarabine, 19.4% (*n* = 59) were given daunorubicin, and 19.1% (*n* = 58) received azacitidine. A total of 75% (*n* = 30) of patients with MDS received treatment prior to venetoclax, with 77.2% (*n* = 27) receiving azacitidine, 14.3% (*n* = 5) receiving decitabine, and 8.5% (*n* = 3) receiving other treatment options. A total of 90.7% of patients with AML and 97.5% of patients with MDS did not undergo HSCT prior to venetoclax treatment. A total of 84.5% (*n* = 136) of patients with AML received combination therapy with azacitidine, 13.7% (*n* = 22) with decitabine, and only 1 patient received monotherapy. A total of 67.5% (*n* = 27) of patients with MDS received combination therapy with azacitidine, 22.5% (*n* = 9) with decitabine, and 10% (*n* = 4) received monotherapy.

The mean number of cycles administered during venetoclax treatment is higher in patients with MDS (7.10 ± 4.57). The duration of antifungal prophylaxis is also higher in patients with MDS (392.63 ± 822.63). The mean blast rate before venetoclax treatment in patients with AML is 37.35 ± 24.27 (Table 2).

A total of 90.7% (*n* = 146) of patients with AML had a hematologic response assessment performed on bone marrow samples. Among these patients, 43.2% had the assessment performed in the fourth cycle. This assessment was conducted as follows: bone marrow aspiration in 115 patients, bone marrow biopsy in 69 patients, flow cytometry on bone marrow samples in 57 patients, and routine blood counts in 31 patients. Routine blood counts were performed to monitor hematologic recovery. A total of 80% (*n* = 32) of patients with MDS had a hematologic response assessment performed. Among these patients, 46.9% had the assessment performed in the fourth cycle and 37.5% in the first cycle. This assessment was conducted as follows: bone marrow aspiration in 27 patients, bone marrow biopsy in 24 patients, flow cytometry in 14 patients, and routine blood counts in 12 patients.

Hematologic response in patients with AML was recorded as complete remission with incomplete blood count recovery (CRI) in 31.5% (*n* = 46) and complete remission (CR) in 22.6% (*n* = 33). In patients with MDS, these rates were 37.5% (*n* = 12) for CRI and 25% (*n* = 8) for CR. The number of patients with AML who achieved remission was 92 (57.1%), while 24 patients with MDS (60%) achieved remission. The venetoclax dose administered until remission was 400 mg for 58.7% (*n* = 54) of patients with AML and 200 mg for 58.3% (*n* = 14) of patients with MDS. The venetoclax dose was not reduced in 83.7% (*n* = 77) of patients with AML and 87.5% (*n* = 21) of patients with MDS. Concurrent medication was used in six patients with AML and one patient with MDS. Cytopenia development was observed in eleven patients with AML and two patients with MDS. In the post-remission period, 29.3% (*n* = 27) of patients with AML received 400 mg, 27.2% (*n* = 25) received 100 mg, 58.3% (*n* = 14) of patients with MDS received 200 mg, and 29.2% (*n* = 7) received 100 mg of venetoclax. A total of 65% (*n* = 26) of patients with MDS received treatment in 14-day cycles. The reasons for administering venetoclax to patients with AML were as follows: 29.2% due to relapse–refractory status after HMA-LDAC, 28.6% due to relapse–refractory status after intensive chemotherapy, 26.1% due to first-line use, and 8.1% as a bridge to HSCT relapse after HSCT. The reasons for administering venetoclax to patients with MDS were: 67.5% due to relapse–refractory status after HMA-LDAC, 27.5% due to first-line use, and 2.5% as a bridge to HSCT and relapse after HSCT. The mean duration until remission was higher in patients with AML (17.84 ± 18.73) (Table 3).

A total of 37.9% of patients with AML and 27.5% of patients with MDS received antifungal prophylaxis. More than 54% of the patients were treated with Posaconazole. Among those receiving antifungal treatment, the venetoclax dose was 100 mg for 49.2% (*n* = 30) of patients with AML and 200 mg for 31.1% (*n* = 19). For patients with MDS, 54.5% (*n* = 6) received 200 mg and 45.5% (*n* = 5) received 100 mg. In treatment management, 65.2% (*n* = 105) of patients with AML and 70% (*n* = 28) of patients with MDS used Granulocyte Colony-Stimulating Factor (G-CSF). G-CSF was used post-remission in 71.4% (*n* = 40) of patients with AML and 66.7% (*n* = 8) of patients with MDS. More than 60% of both AML and patients with MDS were hospitalized. The percentage of adverse events leading to treatment discontinuation among those with grade 3–4 toxicity is low: 26.7% for patients with AML (*n* = 43) and 15% for patients with MDS (*n* = 6). Adverse events requiring dose modification occurred in 47.2% (*n* = 76) of patients with AML and 37.5% (*n* = 15) of patients with MDS. Among these, dose reduction (AML 44.7%; *n* = 34, MDS 53.3%; *n* = 8) and treatment delay (AML 30.3%; *n* = 23, MDS 40%; *n* = 6) were the most commonly applied approaches. Relapse development was not observed in 84.5% (*n* = 136) of patients with AML and 92.5% (*n* = 37) of patients with MDS.

The mortality rate was 54.7% (*n* = 88) in patients with AML, while it was 42.5% (*n* = 17) in patients with MDS. The duration of illness was found to be higher in patients with MDS (25.82 ± 19.78). The total number of hospital admission days (43.39 ± 36.81) and the follow-up period (17.60 ± 13.09 months) were higher in patients with AML (Table 2).

**Figure 1:**
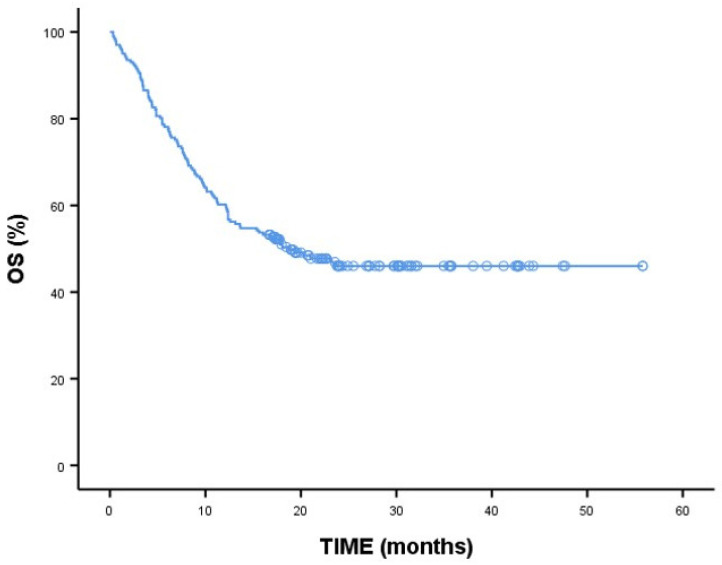
Two-year overall survival (OS) analyses. Kaplan–Meier curve; long-rank test; *p* < 0.05 is statistically significant.

As shown in Table 4, the 2-year overall survival rate is 46%. The 2-year OS results are shown in Figure 1. The median overall survival was determined to be 18.73 months. For patients with AML, this rate is 44.1% with a mean survival of 17.33 months, while for patients with MDS, it is 52.7%. When comparing diagnosis groups, no significant difference was found between AML and MDS (*p* = 0.278). No difference was observed between the groups in terms of response assessment (*p* = 0.138), and the number of cycles of venetoclax treatment did not show a statistically significant difference in median overall survival (months) across the groups (*p* = 0.241).

In the AML group, the median overall survival was determined to be 17.33 months (range of 10.21–24.44 months). In the AML group, overall survival was not statistically significantly associated with response (*p* = 0.454) or the number of cycles of venetoclax treatment (*p* = 0.562). Median overall survival was also not statistically significantly associated with responses (*p* = 0.174) in the AML group. For the MDS group, the median overall survival could not be calculated (Table 5).

**Figure 2:**
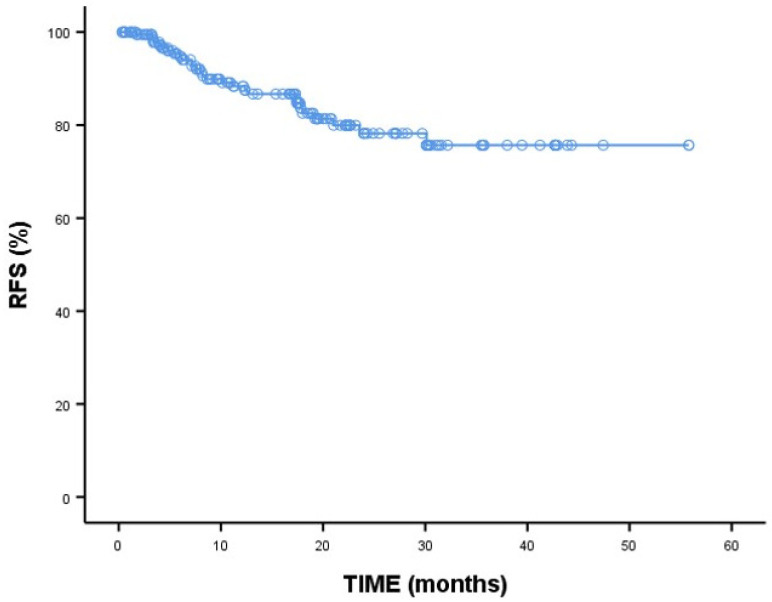
Two-year recurrence-free survival (RFS) analyses. Kaplan–Meier curve; long-rank test; *p* < 0.05 is statistically significant.

The overall disease-free survival rate for patients is 78.2%. As shown in Table 6, the median recurrence-free survival (RFS) in months could not be obtained. The 2-year RFS rate is 90.9% in patients with MDS and 75.5% in patients with AML. The 2-year RFS results are shown in Figure 2. There is no significant difference between the two groups (*p* = 0.222). Median RFS (months) was not statistically significant according to response (*p* = 0.883). However, the number of cycles of venetoclax treatment was statistically significantly associated with median RFS (months) across the groups (*p* = 0.004). The 2-year survival rate for patients who received venetoclax in the second and third cycles was significantly higher compared to those who received it in the first cycle.

In the AML group, median RFS (months) was not statistically significant according to response (*p* = 0.820). However, median RFS (months) was statistically significantly associated with the number of cycles of venetoclax treatment (*p* = 0.001). In the MDS group, the general median RFS (months) could not be obtained (Table 7).

## 4. Discussion

Current treatments for AML/MDS are both limited in number and efficacy [13,15,16,17]. Since the FDA approved venetoclax for AML, its off-label use in combination with HMA for AML/MDS has become more common, but there is limited data on its efficacy. To address this, we conducted a retrospective analysis of 201 patients receiving this treatment to assess real-world outcomes and its potential impact on overall survival (OS).

In this study, patients with AML are on average over 63 years old, while patients with MDS have a mean age of 70. In both patient groups, over 55% are male. Due to the mean age of the patients included in this study, HSCT treatment was applied to a limited number of patients. Venetoclax was administered in combination with azacitidine to 84.5% of patients with AML and 67.5% of patients with MDS. The reasons for administering venetoclax to patients with AML include relapse–refractory status after HMA-LDAC and intensive chemotherapy (29.2%, 28.6%) and first-line use (26.1%). Hematologic response was observed more frequently at the end of the fourth cycle and less frequently at the end of the first and second cycles. FLT3 mutations were observed in only 10.6% of patients with AML. The relapse rate in patients with AML is approximately 15%.

Aldoss et al. reported a higher overall response rate of 64% in 33 adult patients with relapsed/refractory AML, with the best response achieved after a median of two cycles. Of these patients, 60% had prior exposure to HMA [17]. DiNardo et al. reported on 43 patients with R/R AML and related myeloid malignancies that received venetoclax in combination with low-intensity therapies. This cohort included older (median age 68 years) and heavily pretreated patients (median number of prior treatment lines = 3), and most patients (77%) had previous exposure to HMA. The overall response in this cohort was rather low (21%) with 12% of the patients attaining a CR/CRi [18]. Better overall responses were reported by Aldoss et al., who reported on 33 adult patients with R/R AML (median age 62 years), 60% of whom had previous exposure to HMA. The overall response rate was 64%, and the best response was achieved after a median of two cycles [17]. In this study, overall, the 2-year survival rate is 46% and 18.73 months. The overall CR/CRi rate for patients with AML is 49.1%, with the best response achieved during the second cycle of venetoclax treatment.

The optimal dose and duration of venetoclax treatment in MDS are still under investigation, particularly for elderly patients. Although a phase-2 dose of 400 mg daily for a 28-day cycle in combination with azacitidine has been established, it is based on limited data, and further research is needed [19].

Current clinical trials are evaluating the effectiveness of venetoclax and azacitidine in the treatment of R/R HR-MDS. Preliminary results from a phase 1b trial indicate that venetoclax-based combination therapy has a median response time of 1.2 months, an ORR of 40%, and a 12-month OS estimate of 65%. Additionally, a retrospective study of patients with HR-MDS treated with venetoclax and an HMA revealed a 59% mCR rate, with 62% of responders undergoing allo-SCT [20]. This suggests that venetoclax-based combination therapy could serve as a bridge to allo-SCT in the future [21]. In this study, in patients with MDS, the overall CR/CRi rate is 50% and the 2-year survival rate is 52.7%. Prior to venetoclax treatment, 77.6% of patients with AML and 75% of patients with MDS had received HMA therapy.

The phase III study for first-line treatment of patients with AML who are not able to tolerate intensive treatment uses 400 mg as a daily dose [10]. Exposure–response data support a 400 mg venetoclax daily dose as reasonable in combination with HMA [22]. In this study, remission was achieved by 92 patients with AML (57.1%) and 24 patients with MDS (60%). The dose of venetoclax administered until remission was 400 mg for patients with AML and 200 mg for patients with MDS. For MDS treatment, 65% of the cases received treatment for 14 days within a 28-day cycle.

When evaluating AML/MDS together, overall the RFS rates after two and three cycles of venetoclax treatment (83.9% and 81.5%, respectively) were significantly higher compared to one cycle (44.6%). In this study, the 2-year RFS rate is 78.2%. When assessed separately, the 2-year RFS rate was 75.5% for patients with AML and 90.9% for patients with MDS. In patients with AML alone, the RFS rates after two and three cycles of venetoclax treatment (86.2% and 78.9%, respectively) were significantly higher compared to one cycle (39.5%). In a study with 23 patients and a 2-year follow-up period, febrile neutropenia was commonly observed (78%). The average hospital stay was found to be 13 days [23]. In our study, the incidence of neutropenia in patients with AML/MDS is around 30%. More than 65% of these patients received G-CSF after remission. Neutropenia developed in 40 patients with AML and 8 patients with MDS following remission. In another study, the primary safety concern was hematologic, with neutropenia being the most commonly observed side effect in trials where venetoclax was used for AML treatment. In that study, 68.8% of patients had grade 3 or higher neutropenia. Nearly half of these patients required GCSF support for grade 3 or higher neutropenia [24]. In the study, the proportion of patients with grade 3 or 4 neutropenia was 26.7% for AML and 15% for MDS. In our study, the mean hospital stay was 43 days for patients with AML and 23 days for patients with MDS. The reasons for hospitalization were primarily events requiring dose modifications. The approach taken for these patients was dose reduction.

In the treatment with venetoclax, the median survival rate for patients with MDS was found to be higher compared to patients with AML. Venetoclax exhibits a low toxic profile, a low relapse rate, and very rare adverse events leading to treatment discontinuation. These results suggest that venetoclax treatment may offer significant clinical responses in both disease groups. Overall, this study highlights the potential of venetoclax to achieve higher overall survival rates when used in combination therapies. Suggestions for future prospective studies, particularly regarding optimal dosing schedules or patient populations that may benefit most from venetoclax treatment, are warranted. Validation of these findings in prospective studies and clinical trials is necessary.

## Figures and Tables

**Table 1:** Patient characteristics.

	Diagnosis	
Variables	AML *n* = 161	MDS *n* = 40
Age, mean ± SD	63.53 ± 15.30	70.12 ± 10.21
Gender, *n* (%)		
Male	94 (58.4)	23 (57.5)
Female	67 (41.6)	17 (42.5)
AML Type, *n* (%)		
De novo	115 (71.4)	
Secondary	46 (28.6)	
FLT3 Mutation, *n* (%)		
Absent	144 (89.4)	
Present	17 (10.6)	
ELN, 2017 Cytogenetics, *n* (%)		
Good	10 (6.2)	
Intermediate	93 (57.8)	
Poor	36 (22.4)	
Unknown	22 (13.7)	
MDS Subtype, *n* (%)		
Unclassified MDS with excess blasts refractory anemia		33 (82.5)
Multiple dysplastic and ring sideroblasts refractory cytopenia		2 (5.0)
Chronic myelomonocytic leukemia and juvenile myelomonocytic leukemia		2 (5.0)
Ring sideroblasts refractory anemia		1 (2.5)
MDS IPSS-R Score, *n* (%)		
Low		4 (10.0)
Intermediate		26 (12.9)
High		10 (25.0)
R-IPSS Risk, *n* (%)		
Intermediate		3 (7.5)
High		28 (70.0)
Very high		9 (4.5)

Abbreviations: AML = acute myeloid leukemia; MDS = myelodysplastic syndrome; IPSS-R = revised international prognostic scoring system; FLT3 = Fms-like tyrosine kinase 3; ELN = European LeukemiaNet.

**Table 2:** Treatments administered to the patients.

	Treatment	
	AML *n* = 161	MDS *n* = 40
Pre-treatment before venetoclax, *n* (%)		
Present	125 (77.6)	30 (75)
Absent	36 (22.4)	10 (25)
Treatments received before venetoclax, *n* (%)		
Cytarabine	89 (29.3)	
Idarubicin	24 (7.9)	
Daunorubicin	59 (19.4)	
Azacitidine	58 (19.1)	27 (77.2)
Decitabine	5 (1.6)	5 (14.3)
Mitoxantrone	12 (3.9)	
Etoposide	16 (5.2)	
Busulfan	3 (0.9)	
Cyclophosphamide	13 (4.2)	
Other	24 (7.9)	3 (8.5)
HSCT performed before venetoclax treatment, *n* (%)		
Yes	15 (9.3)	1 (2.5)
No	146 (90.7)	39 (97.5)
AML venetoclax application, *n* (%)		
Monotherapy	1 (0.6)	4 (10.0)
Combined with azacitidine	136 (84.5)	27 (67.5)
Combined with decitabine	22 (13.7)	9 (22.5)
MDS treatment usage in 28-day cycles, *n* (%)		
14 days		26 (65.0)
21 days		8 (20.0)
28 days		6 (15.0)
Use G-CSF in treatment management		
Yes	56 (34.8)	12 (30)
No	105 (65.2)	28 (70)
G-CSF timing		
Pre-remission	16 (28.6)	4 (33.3)
Post-remission	40 (71.4)	8 (66.7)
Relapse development, *n* (%)		
Absent	136 (84.5)	37 (92.5)
Present	25 (15.5)	3 (7.5)
Mortality, *n* (%)		
Alive	73 (45.3)	23 (57.5)
Deceased	88 (54.7)	17 (42.5)
Disease duration, mean ± SD (month)	19.83 ± 17.91	25.82 ± 19.78
Baseline blast rate before venetoclax, mean ± SD	37.35 ± 24.27	
Number of cycles administered during venetoclax treatment, mean ± SD	5.66 ± 3.95	7.10 ± 4.57

Abbreviations: AML = acute myeloid leukemia; MDS = myelodysplastic syndrome; HSCT = Hematopoietic Stem Cell Transplantation; G-CSF: Granulocyte Colony-Stimulating Factor.

**Table 3:** Hematologic response assessment.

Hematologic Response Assessment, *n* (%)	AML *n* = 161	MDS *n* = 40
Yes	146 (90.7)	32 (80)
No	15 (9.3)	8 (20)
Bone marrow biopsy	69 (47.3)	24 (75)
Bone marrow aspiration	115 (78.8)	27 (84.4)
Routine blood count	31 (21.2)	12 (37.5)
Flow cytometry	57 (39)	14 (43.7)
Timing		
End of Cycle 1	37 (25.3)	12 (37.5)
End of Cycle 2	39 (26.7)	4 (12.5)
End of Cycle 3	3 (2.1)	1 (3.1)
End of Cycle 4	63 (43.2)	15 (46.9)
End of Cycle 6	4 (2.7)	0 (0)
Hematologic response, *n* (%)		
CR	33 (22.6)	8 (25)
CRI	46 (31.5)	12 (37.5)
MLFS	18 (12.3)	4 (12.5)
PR	26 (17.8)	3 (9.4)
SD	10 (6.8)	3 (9.4)
PD	13 (8.9)	2 (6.3)
Remission, *n* (%)		
Yes	92 (57.1)	24 (60)
No	69 (42.9)	16 (40)
Venetoclax dose administered until remission, *n* (%)		
100 mg	17 (18.5)	3 (12.5)
200 mg	16 (17.4)	14 (58.3)
300 mg	5 (5.4)	0 (0)
400 mg	54 (58.7)	7 (29.2)
Venetoclax dose reduced before remission, *n* (%)		
Yes	15 (16.3)	3 (12.5)
No	77 (83.7)	21 (87.5)
Reduced venetoclax dose, *n* (%)		
50 mg	2 (13.3)	0 (0)
70 mg	2 (13.3)	0 (0)
100 mg	4 (26.7)	1 (50)
200 mg	7 (46.7)	0 (0)
300 mg	0 (0)	1 (50)
Concurrent medication use, *n* (%)		
Yes	6 (3.7)	1 (2.5)
Cytopenia development, *n* (%)		
Yes	11 (6.8)	2 (5)
Venetoclax dose administered after remission, *n* (%)		
100 mg	25 (27.2)	7 (29.2)
200 mg	23 (25)	14 (58.3)
300 mg	1 (1.1)	2 (8.3)
400 mg	27 (29.3)	1 (4.2)
Discontinued	16 (17.4)	0 (0)
Time to remission, mean ± SD (month)	17.84 ± 18.73	15.54 ± 12.16

AML = acute myeloid leukemia; MDS = myelodysplastic syndrome; CR = complete remission; CRi = complete remission with incomplete hematological recovery; MLFS = morphological leukemia-free state; PR = partial response; SD = stable disease; PD = progressive disease.

**Table 4:** Comparisons of overall survival (OS) for patients.

OS (Months)	2 Years (%)	Median (%95 CI)	*p*
General	46.0	18.73 (-)	
Diagnosis			
AML	44.1	17.33 (10.21–24.44)	0.278
MDS	52.7	- (-)	
Response			
CR	52.9	- (-)	0.138
MLFS	26.5	8.13 (3.88–12.38)	
PR	47.0	23.70 (-)	
SD	14.4	16.80 (10.02–23.57)	
PD	45.0	18.73 (5.35–32.11)	
Cycles of venetoclax treatment			
1	34.3	13.66 (1.34–25.99)	0.241
2	65.2	- (-)	
3 and above	44.7	18.03 (9.87–26.19)	

Kaplan–Meier curve; long-rank test; *p* < 0.05 is statistically significant.

**Table 5:** Comparisons of overall survival (OS) by diagnosis groups.

	AML *n* = 161			MDS *n* = 40		
OS (Months)	2 Years %	Median (%95 CI)	*p*	2 Years %	Median (%95 CI)	*p*
General	44.1	17.33 (10.21–24.44)		52.7	- (-)	
Response						
CR	50.2	- (-)	0.454	65.0	- (-)	0.174
MLFS	33.3	8.46 (1.05–15.88)		-	4.20 (0.00–8.83)	
PR	44.4	12.60 (0.00–31.12)		66.7	- (-)	
SD	13.3	12.26 (0.00–25.23)		-	16.80 (15.57–18.02)	
PD	53.8	- (-)		-	- (-)	
Cycles of venetoclax treatment						
1	35.7	12.36 (2.28–22.45)	0.562			
2	57.9	- (-)				
3 and above	43.0	15.60 (7.11–24.08)				

Kaplan–Meier curve; long-rank test; *p* < 0.05 is statistically significant.

**Table 6:** Comparisons of recurrence-free survival (RFS) for patients.

RFS (Months)	2 Years %	Median (%95 CI)	*p*
General	78.2	- (-)	
Diagnosis			
AML	75.5	- (-)	0.222
MDS	90.9	- (-)	
Response			
CR	82.3	- (-)	0.883
MLFS	73.1	- (-)	
PR	74.3	- (-)	
SD	83.3	- (-)	
PD	83.1	- (-)	
Cycles of venetoclax treatment			
1	44.6	19.20 (14.30–24.09)	0.004
2	83.9	- (-)	
3 and above	81.5	- (-)	

Kaplan–Meier curve; long-rank test; and *p* < 0.05 is statistically significant.

**Table 7:** Comparisons of recurrence-free survival (RFS) by diagnosis groups.

	AML *n* = 161			MDS *n* = 40		
RFS (Months)	2 Years %	Median (%95 CI)	*p*	2 Years %	Median (%95 CI)	*p*
General	75.5	- (-)		90.9	- (-)	
Response						
CR	80.9	- (-)	0.820			
MLFS	69.6	- (-)				
PR	77.0	- (-)				
SD	77.8	- (-)				
PD	79.5	- (-)				
Cycles of venetoclax treatment						
1	39.5	17.73 (1.09–33.56)	0.001			
2	86.2	- (-)				
3 and above	78.9	- (-)				

Kaplan–Meier curve; long-rank test; *p* < 0.05 is statistically significant.

## Data Availability

The raw data have been stored at electronic case report form platform of Turkish Hematology Network Grup. These data can be accessable only by the researchers and can be reused as data sources by other researches that have ethical committee approval.
